# Probing the dynamic stalk region of the ribosome using solution NMR

**DOI:** 10.1038/s41598-019-49190-1

**Published:** 2019-09-19

**Authors:** Xiaolin Wang, John P. Kirkpatrick, Hélène M. M. Launay, Alfonso de Simone, Daniel Häussinger, Christopher M. Dobson, Michele Vendruscolo, Lisa D. Cabrita, Christopher A. Waudby, John Christodoulou

**Affiliations:** 1 Institute of Structural and Molecular Biology, UCL and Birkbeck College London, Gower Street, London, WC1E 6BT UK; 2 Department of Life Sciences, Imperial College London, South Kensington Campus, London, SW7 2AZ UK; 3 Department of Chemistry, University of Basel, St. Johannsring 19, 4056 Basel, Switzerland; 4 Centre for Misfolding Disease, Department of Chemistry, University of Cambridge, Lensfield Road, Cambridge, CB2 1EW UK

**Keywords:** Structural biology, Structure determination, Solution-state NMR, Molecular dynamics

## Abstract

We describe an NMR approach based on the measurement of residual dipolar couplings (RDCs) to probe the structural and motional properties of the dynamic regions of the ribosome. Alignment of intact 70S ribosomes in filamentous bacteriophage enabled measurement of RDCs in the mobile C-terminal domain (CTD) of the stalk protein bL12. A structural refinement of this domain using the observed RDCs did not show large changes relative to the isolated protein in the absence of the ribosome, and we also found that alignment of the CTD was almost independent of the presence of the core ribosome particle, indicating that the inter-domain linker has significant flexibility. The nature of this linker was subsequently probed in more detail using a paramagnetic alignment strategy, which revealed partial propagation of alignment between neighbouring domains, providing direct experimental validation of a structural ensemble previously derived from SAXS and NMR relaxation measurements. Our results demonstrate the prospect of better characterising dynamical and functional regions of more challenging macromolecular machines and systems, for example ribosome–nascent chain complexes.

## Introduction

In recent years, X-ray crystallography and cryo-electron microscopy (cryo-EM) have elucidated the details of high-resolution structures of ribosomes, revealing intricate mechanistic information about their function during the translation process^1,2^. In parallel, NMR-based observations of nascent polypeptide chains emerging from the ribosome are providing unique structural and mechanistic insights into co-translational folding processes^3–5^. In order to develop further solution-state NMR spectroscopy as a technique for structural studies of dynamic regions within large complexes, we have explored the measurement of residual dipolar couplings (RDCs) within intact ribosomes, focusing in particular on the mobile bL12 protein from the GTPase-associated region (GAR) of the prokaryotic 70S ribosome. RDCs have been used to characterise other macromolecular machines and assemblies, including HIV-1 capsid protein, bacterial Enzyme I, and the 20S proteasome^6–8^. These developments are particularly relevant as macromolecular complexes tend to exhibit a wide variety of functional motions that are challenging to characterise by methods such as X-ray crystallography or cryoelectron microscopy.

**Figure 1:**
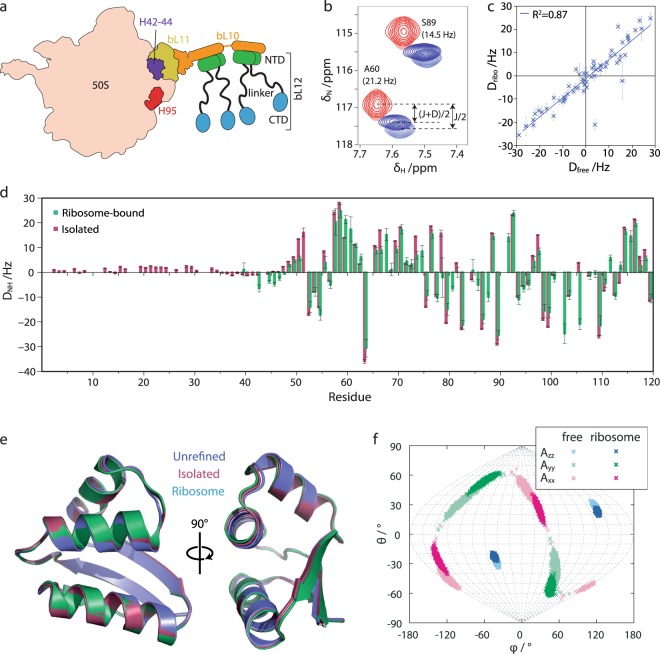
RDC measurement and analysis of ribosome-bound and free bL12 aligned in Pf1 phage. (**a**) Schematic illustration of the 50S subunit of the bacterial ribosome illustrating the position and composition of the stalk region. (**b**) Excerpt from ^1^H, ^15^N HSQC (red) and TROSY (blue) spectra of the ribosome-bound bL12 CTD in isotropic and aligned conditions (light and dark colouring, respectively), acquired at 700 MHz and 298 K. (**c**) Correlation plot of measured amide RDCs between free and ribosome-bound bL12. The error bars represent the uncertainties in the measured RDCs, as estimated from the signal-to-noise ratios and linewidths of the spectral peaks. (**d**) Amide RDCs measured for ribosome-bound and isolated bL12. (**e**) Refinement of the bL12 template structure (PDB code:1rqu) using observed RDCs for isolated and ribosome-bound bL12. (**f**) Sanson-Flamsteed projection of the principal axes of the alignment tensor calculated for free and ribosome-bound bL12.

The GAR is a highly conserved region of both prokaryotic and eukaryotic ribosomes, and is so named to reflect its role in both the recruitment and the stimulation of the GTPase activity of several auxiliary factors associated with the key steps of protein synthesis: initiation (initiation factor 2, IF2), elongation (elongation factors EF-Tu and EF-G) and termination (release factor 3, RF3)^9^. The prokaryotic GAR includes helices 42–44 and 95 of the 23S rRNA, and the ribosomal proteins bL10, bL11 and bL12 (Fig. 1a). bL12, the focus of the present work, is a 120 residue dimeric protein consisting of an N-terminal dimerisation domain (NTD), which binds to the extended bL10 helix of the core ribosome particle, and a C-terminal domain (CTD), which interacts with GTPase substrates to facilitate their recruitment to the ribosome. The bL12 CTD interacts with four major translational GTPases, IF2, EF-Tu, EF-G and RF3, through a highly conserved and positively charged binding site in helices 4 and 5, identified through NMR mapping and mutagenesis^10–14^. GTPase binding has been found by mutagenesis to occur through the G4–G5 helix in the G domain of IF2 (and, by homology, likely also via the G domain in other GTPases), which presents a complementary negatively charged surface and results in extremely rapid binding driven by favourable electrostatics^14^. The CTD is mobile in solution, being separated from the NTD by a flexible hinge region, and is absent in structures of ribosomes determined by X-ray crystallography and cryoelectron microscopy.

bL12 is unique among ribosomal proteins in being present in multiple copies: typically four or six monomers are present, although some cyanobacteria may contain eight^15^. In the particular case of the *E*. *coli* ribosome studied here, four copies are present^16^. However, while bL12 is essential for protein synthesis^17^, not all copies are needed. While variant ribosomes containing only one bL12 dimer show less than 50% of the protein synthesis activity of wild-type ribosomes^18^, engineered variants in which only one CTD is present per bL12 dimer have approximately 80% activity related to wild-type ribosomes^19^. These observations suggest therefore that only one CTD may be functioning within each dimer at any one time.

Aside from the CTD, the flexible hinge region of bL12 is also essential for ribosome activity^20^. However, this flexibility, which has been proposed to facilitate the recruitment of translation factors from the cellular space to the 30S/50S interface^9,21,22^, has precluded the observation and structure determination of the hinge region and CTD by cryo-EM and X-ray crystallography. Solution-state NMR spectroscopy is therefore uniquely suited to the structural characterisation of this region. Indeed, ^1^H,^15^N-correlation spectra of 70S ribosomes were found to contain resonances arising almost exclusively from the bL12 CTD^23–25^. No chemical shift perturbations were observed between the CTD in isolation and when bound to the ribosome, implying that the domain structure is not perturbed by ribosome binding. However, analyses of resonance intensities showed that only two of the four CTDs have sufficient mobility to be detectable by solution-state NMR, indicating that significant dynamical heterogeneity exists within the stalk region^24,25^. Futhermore, ^15^N nuclear spin relaxation measurements were used to characterise the anisotropic rotational diffusion of the 50S ribosome-bound CTD, from which it was observed that the diffusion tensor of the CTD was rotated 59.7° relative to the inertia tensor of the domain, indicating that the motion of the domain is perturbed by the presence of the hinge region and potentially also interactions between the CTD and the ribosome surface^24^. Within isolated bL12 dimers, an NMR analysis of secondary ^1^H^α^ chemical shifts within the hinge region indicated a weak propensity for beta-sheet structure, indicating that the hinge may not be completely flexible^26^. Further measurements of NMR relaxation and small-angle x-ray scattering (SAXS) were combined with molecular modelling to create an optimized structural ensemble of the bL12 dimer, which also indicated that the hinge region was not as flexible as would be expected from a random coil, and that the orientations of the NTD and CTD were instead partially coupled, with an order parameter of *S*^2^ = 0.17^27^.

In this work, we have continued our NMR investigations of bL12 by measuring residual dipolar couplings (RDCs) for this protein in the spectrum of intact 70S ribosomes, in order to characterize both the structure of the CTD and the dynamical properties of the hinge region. In particular, we have sought to characterise the extent to which the orientation of the bL12 CTD is coupled to the (ribosome-bound) NTD, and so test the accuracy of the optimized structural ensemble described above^27^. RDCs can be measured under weakly anisotropic solution conditions and report on both the local structure of the protein and the overall orientation of domains with respect to a laboratory frame of reference^28,29^. As such, they are useful sources of distance-independent structural information that are highly complementary to chemical shifts, scalar couplings and NOEs. Anisotropy can be induced by a variety of steric and electrostatic methods including liquid crystals, bicelles, bacteriophage and stretched polyacrylamide gels^30^.

## Results

### RDCs measurement of the intact 70S ribosome

We tested the alignment of samples of ribosomes in Pf1 bacteriophage and C_12_E_5_ PEG/hexanol mixtures^31^. The latter induced inhomogeneous alignment (Methods), but strong alignment (12.3 Hz ^2^H quadrupolar splitting) was obtained in Pf1 bacteriophage and this system was therefore used for further measurements (see Supplementary Fig. 1). Amide RDCs (*D*_NH_) were determined for ribosome-bound bL12 by measurement of ^15^N frequency differences between amide resonances in ^1^H, ^15^N HSQC and TROSY spectra (Fig. 1b)^32^. We found that this approach provided the greatest precision in values of RDCs when compared with alternative methods such as the in-phase/anti-phase approach (see Supplementary Fig. 2). Measurements of additional couplings to C’ and Cα atoms were also explored, but the sensitivity was not sufficient to obtain precise results. The integrity of ribosomal samples was monitored with ^1^H stimulated-echo diffusion measurements, acquired in an interleaved fashion with HSQC and TROSY experiments (see Supplementary Fig. 3)^33^.

Despite the 2.4 MDa molecular-weight of the 70S ribosome, and the low sample concentration (10 µM), RDCs could be obtained for the entire CTD of ribosomal bL12 (bL12_ribo_), with an RMS uncertainty ($\langle {\sigma }_{D}^{2}{\rangle }^{1/2}$) of 1.8 Hz relative to a range of ± 30 Hz (Fig. 1c,d and Supplementary Tables S1, S2). Despite the multiple copies of bL12 on the ribosome, of which two are observable by solution-state NMR^24,25^, for all residues only single resonances were resolved, in both the presence and absence of bacteriophage, with no discernable distortions in lineshapes. This indicates either that the conformation and alignment of domains is effectively averaged over the *ca*. 100 ms timescale (1/*D*_NH_) of the residual dipolar coupling, or that for all residues the difference in residual dipolar couplings between copies is much smaller than the ^15^N TROSY linewidth (11 ± 2 Hz, s.d.). As only a single set of resonances is observed for bL12 (i.e. isotropic chemical shifts are also averaged between multiple copies), the former possibility seems more likely, but as in the latter case any differences that exist must be smaller than the ^15^N linewidth, they would also be smaller than the experimental uncertainty and hence unlikely to affect the conclusions of the present study. RDCs in the CTD of isolated bL12 (bL12_free_) were of a similar magnitude to those of the domain on the ribosome (Fig. 1c), but alignment of the isolated NTD (whose resonances are unobservable in the ribosome-bound form) appeared to be significantly weaker, with RDC values of only a few Hz (Fig. 1d). These small RDCs may be due to the weaker electric dipole moment of the NTD relative to the CTD, which contains a highly charged binding site for GTPase substrates^14^, leading to weaker electrostatic interactions between the NTD and the phage^34^ and hence weaker alignment relative to the CTD.

### Structure refinement

The measured RDCs for the CTD were fitted by singular-value-decomposition (SVD) to a template structure (PDB code: 1rqu^35^) to determine the alignment tensor, *A*^36^. The corresponding back-calculated RDCs showed good agreement with observed RDCs for both bL12_ribo_ and bL12_free_ (with *Q* factors of 0.31 and 0.26 respectively for secondary structure elements, Table S1 and Figure S1), although for both datasets the RMS deviation (RMSD) between measured and back-calculated RDCs was larger than the RMS uncertainty in the measurements (Supplementary Table S1). Thus, while the structures of both bL12_ribo_ and bL12_free_ CTD are clearly similar to the template structure, some additional information appears to be present. This finding suggests the possibility of using the RDC data to derive improved structures for both bL12_ribo_ and bL12_free_.

Where RDCs have been measured for multiple internuclear vectors, or in multiple alignment media, detailed structure or ensemble structure calculations may be performed by minimization of a target energy function^37^ or using restrained molecular dynamics simulations^38^. Ensemble structures are particularly effective for representing flexible or disordered regions of a domain such as loops, and their analysis may be helpful in reducing bias in fitting alignment tensors, although a single structure is also generally regarded as sufficient for this purpose provided that flexible regions are excluded^30^. As in the present situation N-H RDCs could only be measured in a single alignment medium, to avoid overfitting we limited ourselves to refinement of secondary structure elements in a single structure (Methods). This procedure resulted in small changes for both bL12_ribo_ and bL12_free_ structures (Fig. 1e), with Cα RMSDs (of all residues, relative to the template structure) of 0.35 Å and 0.52 Å respectively, while *Q* factors for secondary structure elements improved slightly, to 0.24 and 0.18 respectively (Supplementary Table S1). The RMSD between the measured and back-calculated RDCs remained larger than the experimental error, indicating that some structural noise relative to the true structure is still present even in the refined structure. However, further structural refinement is not feasible with N-H RDCs alone, due to the risk of overfitting and potentially distortion of the peptide bond geometry. The differences between the template and refined structures are not large, and neither is the difference between free and ribosome-bound forms, but this is perhaps to be expected given the previously observed absence of chemical shift perturbations in the CTD between free and ribosome-bound forms^24,25^. We also note that the original isolated CTD structure^35^ was determined from a more comprehensive experimental dataset than the single set of N-H RDCs we have measured here – in analogy with data available from ribosome-bound measurements – and so is still likely to represent the more accurate structure. Nevertheless, the calculations demonstrated here are an important step towards the use of RDCs for structural refinement in more complex systems, such as disordered ribosome–nascent chain complexes, for which much less is known of the ribosome-associated structure *a priori*^3,4^.

### Alignment tensor analysis of bL12 CTD

Following refinement, we used the resulting structures to re-fit alignment tensors to the observed RDCs and so compare the preferred orientations of free and ribosome-bound bL12. The orientations of the principal axes of the alignment tensors can be visualised in a Sanson-Flamsteed projection (Fig. 1f). Numerical uncertainties were calculated using a Monte Carlo approach^39^ that accounts for both experimental errors in the measured RDCs and residual structural noise in the refined template structure (which is of particular importance here as a single refined structure rather than an ensemble is used for this analysis). We note that while individual differences between free and ribosome-bound RDCs may be comparable to the experimental uncertainty, the global analysis of such measurements across multiple residues can nevertheless identify significant differences, and indeed we observe a small difference in the orientations of the *A*_*xx*_ and *A*_*yy*_ axes (Fig. 1f), with a corresponding difference between the 5D angles^40^ of the two tensors of 13.8 ± 7.6°. This result indicates that the orientation of the bL12_ribo_ CTD is influenced by the presence of the core ribosome particle, demonstrating that the bL12 CTD is not completely independent.

The influence of the ribosome on the orientation of the bL12 CTD could be due to incomplete flexibility of the linker region of bL12, or could be the result of transient interactions between the bL12 CTD and the ribosome core particle. We have previously shown that measurements of transferred transverse relaxation provide a sensitive probe of even weak interactions between nascent chains and the surface of the core ribosome particle^4^. However, in the case of the bL12 CTD, given that the observed ^15^N transverse relaxation rates of the ribosome-associated CTD (17 ± 7 Hz, s.d.) are only marginally larger than those of the free CTD (13 ± 2 Hz, s.d.), any transiently bound states must be extremely sparsely populated, and so unable to affect the observed alignment of the domain. Therefore, we focused our attention on the nature of the linker region, and sought to develop measurements of the extent to which it might couple the alignment of the CTD and the core ribosome particle.

### Measurement of alignment propagation with a lanthanide-binding tag

**Figure 2:**
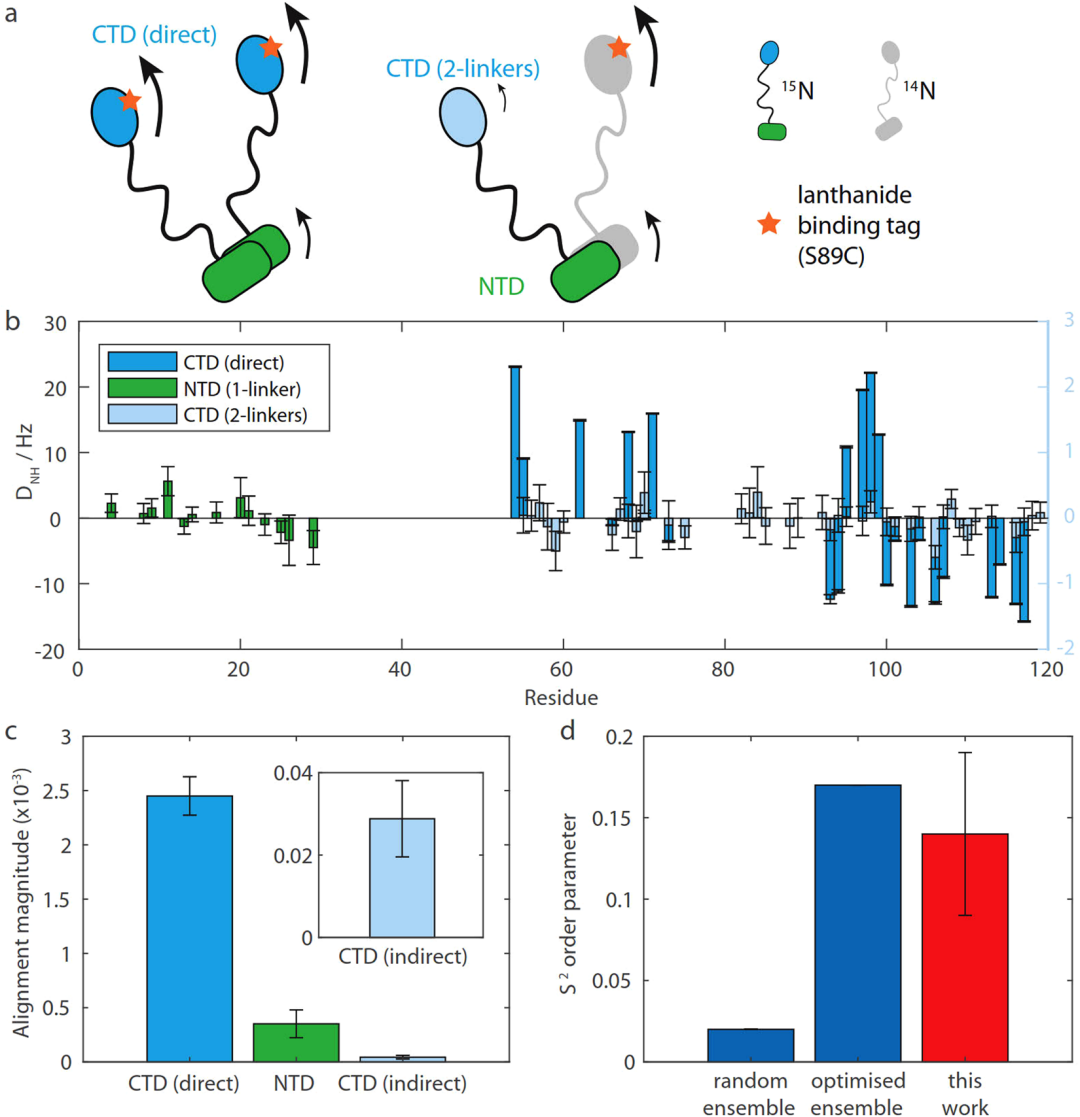
RDC measurement and analysis of bL12 aligned with a paramagnetic lanthanide-binding-tag. (**a**) Mixed ^15^N/lanthanide-binding-tag labelling schemes for measurement of the paramagnetic alignment of domains. (**b**) Amide RDCs measured for directly and indirectly paramagnetically aligned bL12 domains. (**c**) Magnitude of fitted alignment tensors for directly and indirectly aligned bL12 domains. Error bars represents uncertainties derived from the Monte-Carlo “mcDc” routine available in PALES^39^. (**d**) bL12 linker order parameters determined experimentally as a ratio of the alignment magnitude between the CTD (direct) and the NTD (red, with standard error) and calculated from random and SAXS/NMR-optimised ensembles (blue)^27^.

To investigate further the properties of the linker region, and in particular to quantify the extent to which alignment of the CTD and core ribosome particle (including the bound NTD) might be coupled, we investigated the propagation of alignment between domains in isolated bL12 dimers following the attachment of a paramagnetic lanthanide-binding tag (LBT), Tm-DOTA-M8-4R4S^41^, to bL12 domains (Fig. 2a). We initially prepared three cysteine variants of bL12, containing the mutations S15C and S24C (on the surface of the NTD), and S89C (on the surface of the CTD). ^1^H,^15^N HSQC spectra of all three variants showed dispersed resonances with only small chemical shift perturbations relative to the WT protein (Δδ_NH_ < 0.1 ppm), and similar translational diffusion coefficients were measured with ^1^H STE experiments for the WT and all variants (7.1 ± 0.6 × 10^−11^ m^2^ s^−1^ (s.d.)), indicating that the stability of the domains and dimer formation had not been disrupted (Supplementary Figs 5, 6). However, upon attachment of the LBT to S15C and S24C variants, new resonances were observed with ^1^H chemical shifts between 8 and 8.5 ppm, and these were found to overlay with a spectrum of the oxidized WT protein, which has previously been shown to be monomeric^42^ (Supplementary Fig. 6). ^1^H STE diffusion measurements also showed an increase in translational diffusion coefficients for S15C and S24C (to 10.0 ± 0.1 × 10^−11^ m^2^ s^−1^ and 11.6 ± 0.3 × 10^−11^ m^2^ s^−1^ respectively). No such changes were observed in S89C upon attachment of the LBT. We therefore concluded that attachment of the LBT in both S15C and S24C variants disrupted dimer formation, and so only the S89C variant, containing the tag in the CTD, was used for further measurements.

Two samples were prepared using mixed ^15^N/LBT labelling schemes to distinguish between direct and indirect domain alignment: untagged domains within the bL12 dimer will only exhibit alignment if there is propagation of the alignment from the tagged CTD via the linker(s) (Fig. 2a). The directly tagged CTD showed strong alignment (maximum observed RDCs of *ca*. 20 Hz, Fig. 2b and Supplementary Table S2) with RDCs that fitted well to the template structure (*Q* = 0.15). Alignments of the NTD, and of the untagged CTD (separated by two linkers from the LBT-CTD), were weaker but non-zero (Fig. 2b and Supplementary Table S2), indicating that some alignment is propagated through the linker region.

### Analysis of bL12 CTD motion

The alignment strengths of the different domains can be quantified from the magnitude of alignment tensors determined by SVD fitting of the observed RDCs (Fig. 2c). We note that while the relative uncertainties in individual RDC measurements may be large, this global fitting approach, combined with Monte Carlo error analysis, clearly finds that alignment tensor magnitudes are non-zero (Fig. 2c). The ratio of alignment tensor magnitudes between the directly aligned CTD domain, (2.5 ± 0.2) × 10^−3^, and the indirectly aligned NTD, (3.5 ± 1.3) × 10^−4^, then defines an order parameter, *S*^2^ = 0.14 ± 0.05, that describes the degree of coupling taking place through the linker region. This measurement can be compared with previous predictions based upon: (i) a random ensemble with a fully flexible linker, and (ii) an optimised ensemble refined using SAXS and ^15^N nuclear spin relaxation measurements^27^ (Fig. 2d). Our measurements are consistent with the latter ensemble, an interesting feature of which is the anti-correlated extension and retraction of the two CTDs. The linker extension is beyond that expected for a random coil linker, and may facilitate the efficient recruitment of elongation factors from the surrounding cytosol to the ribosome, while the retracted form may promote the activity of recruited CTPases by stabilizing the GTPase on the ribosome^22^ and remaining bound during GTP hydrolysis^43^. In the context of ribosome-bound bL12, these anticorrelated motions may also be associated with the differential observability by solution NMR of two of the four copies of bL12 present on the bL10 stalk^24,25,27^.

## Discussion

In summary, we have shown that RDCs can be successfully measured by solution-state NMR spectroscopy for the flexible bL12 region of the 2.4 MDa 70S ribosome. The analysis of these measurements indicated that, consistent with the observed absence of chemical shift changes between free and ribosome-associated forms^24,25^, the refined structures of bL12_free_ and bL12_ribo_ are very similar (Cα rmsd 0.37 Å). Importantly, this study has laid the foundations for detailed characterization using RDCs of modified bL12 variants on the ribosome, and also for more detailed structural investigations into the co-translational folding of ribosome–nascent chain complexes, for which the structural consequences of ribosomal tethering are at present poorly understood^3,4^. Our analysis of RDCs also indicated that the alignment of the bL12 CTD was weakly perturbed by the presence of the ribosome, either through direct effects of a partially structured hinge region, or through interactions between the CTD and the ribosome surface. As ^15^N relaxation measurements indicated that any such interactions must be extremely weak, we therefore investigated the nature of the interdomain linker in more detail in the absence of the core ribosome particle, and found that specific paramagnetic alignment of domains could be used to measure the coupling between N- and C-terminal domains due to the interdomain linker, which was characterized by an *S*^2^ order parameter of 0.14 ± 0.05. These results validate a previously determined structural ensemble^27^, indicating that the interdomain linker is not fully flexible and instead allows the tethered C-terminal domains to sample a more expanded conformational space, resulting in more efficient recruitment of elongation factors.

## Methods

### NMR sample preparation

Uniformly ^15^N-labeled ribosomes were prepared by growing *E*. *coli* cells in MDG^44^ medium containing ^15^NH_4_Cl as the nitrogen source for 20 hours at 310 K before harvesting. In order to escape stationary phase and to ensure that the ribosomes are not hibernating, the cell pellet was then resuspended in an equal volume of fresh medium and incubated at 37 °C until a steady increase of OD_600_ allowed harvesting at growth phase. The resulting cell pellet was lysed by French press in purification buffer (30 mM HEPES, 12 mM Mg(OAc)_2_, 1 M KOAc, 5 mM EDTA, 2 mM beta-mercaptoethanol (BME), pH 7.6) supplemented with trace of DNAse. The soluble fraction of the lysate was purified with a 35% (w/v) sucrose cushion prepared in purification buffer supplemented with 5 mM ATP. The resulting ribosomal pellet was further purified through a linear density gradient of 10–35% (w/v) sucrose. Fractions containing 70S ribosomes were identified by SDS-PAGE and pure 70S ribosomes were concentrated and buffer exchanged into Tico buffer (10 mM HEPES, 12 mM MgCl_2_, 30 mM NH_4_Cl, 5 mM EDTA, 2 mM BME, pH 7.6). The concentration of ribosomes was determined from the absorbance at 260 nm using the extinction coefficient *ε* = 4.2 × 10^7^ M^−1^cm^−1^ ^33^.

Uniformly ^15^N-labeled bL12 proteins were prepared by transformation into *E*. *coli* cells and growth in M9 minimal medium containing ^15^NH_4_Cl as the nitrogen source at 37 °C. When the OD_600_ reading reached 0.6, expression was induced with 1 mM IPTG for 4 h at 37 °C. The harvested cell pellet was lysed and purified with a sucrose cushion as described above. bL12 protein was purified from the supernatant using a HiTrap Q HP column (loaded in 50 mM Tris-HCl, pH 8.0; elution with a linear gradient of 0–1 M NaCl), followed by a HiTrap Phenyl HPcolumn (loaded in 25 mM Na_2_HPO_4_, 1.7 M (NH_4_)_2_SO_4_, pH 7.5; elution with a reverse gradient of 1.7–1 M (NH_4_)_2_SO_4_) and finally size exclusion chromatography with an Superdex-200 column (50 mM Na_2_HPO_4_, 100 mM KCl, pH 7.0). The concentration of bL12 protein was determined using the bicinchoninic acid (BCA) protein assay reagent (Pierce). Unlabeled bL12 proteins were prepared the same apart from using unlabeled NH_4_Cl in the growth media.

NMR sample concentrations were 10 µM for the ribosomal particle and 50 µM for isolated bL12. To measure RDCs on the 70S ribosome particle, aligned 70S ribosome samples were prepared by addition of 15 mg ml^−1^ Pf1 filamentous phage (ASLA). For isolated bL12, alignment was achieved by addition of 6.5 mg ml^−1^ filamentous phage. The homogeneity of the resulting anisotropic liquid crystalline medium was confirmed by monitoring the splitting and lineshape of ^2^H solvent signal^45^ (see Supplementary Fig. 1). The other alignment medium tested (C_12_E_5_ PEG and hexanol^31^) was found not to be suitable due to a strong interaction with isolated bL12 protein. In addition, the presence of ribosomes appeared to interfere with the formation of the homogeneously aligned lamellar phase, as judged from the irregular lineshape of the ^2^H solvent signal (see Supplementary Fig. 1). This latter effect is most likely to arise from the diameter of the ribosomal particle being larger than the inter-lamellar spacing at the PEG concentration used.

To induce alignment using the Tm-DOTA-M8-4R4S^41^ lanthanide binding tag (LBT), cysteine mutations were introduced at positions S15, S24 and S89. 2D ^1^H,^15^N correlation spectra and ^1^H STE diffusion measurements were acquired for all variants to assess possible perturbations of structure and dimer formation. For labeling, bL12 (S15C, S24C and S89C) protein was incubated in buffer containing 10 mM DTT and then buffer exchanged into a non-DTT containing buffer on a PD-10 desalting column and reacted with the LBT immediately. The reaction was followed by ^1^H, ^15^N SOFAST-HMQC NMR at 298 K until there was no further change in the spectrum.

### NMR data acquisition

All NMR experiments were recorded at 298 K on a Bruker Avance III 700 MHz spectrometer equipped with a TCI cryoprobe. Data were acquired by recording a repeated series of relatively short (time) experiments in an interleaved manner. This approach allows the use of 1D and diffusion experiments to monitor the ribosome integrity at regular intervals. The final spectra for measurement of coupling constants were obtained by summing the individual experiments over the time period for which the ribosome was deemed to be intact, as assessed by ^1^H STE diffusion experiments^46^. These were recorded using an encoding/decoding gradient length, *δ*, of 4.8 ms, a diffusion delay, Δ, of 400 ms, and gradient strengths that were 5, 35, 65 and 95% of the maximum, 55.57 G cm^−1^. To exclude interfering low molecular weight components, the integrity of ribosome samples was monitored using the intensity ratio between 65% and 95% gradient strengths (see Supplementary Fig. 3). An initial decrease in the diffusion coefficient for the isotropic sample can be attributed to a poor baseline due to the residual water signal (an issue which in the future could be ameliorated by the use of isotope-edited XSTE experiments^47^), as the diffusion coefficients calculated from experiments later in time drops back to *ca*. 2 × 10^−11^ m^2^ s^−1^, which corresponds to intact 70S ribosomes^33^. The slower diffusion observed for the aligned sample is a result of elevated viscosity due to the presence of phage (see Supplementary Fig. 3).

Both in-phase/anti-phase (IPAP) ^1^H-coupled ^15^N-HSQC spectra and the pairwise combination of ^15^N-HSQC/^15^N-TROSY spectra were recorded for the ribosome, and the combination of ^15^N-HSQC/^15^N-TROSY spectra was found to yield slightly smaller relative uncertainties in the calculated RDCs than with the approach where two sub-spectra containing the downfield and upfield lines of the ^15^N doublets are generated by taking the sum and difference between in-phase and anti-phase ^1^H-coupled ^15^N-HSQC spectra (see Supplementary Fig. 2).

Backbone ^15^N *R*_2_ relaxation rates were measured on ribosome-bound and isolated bL12 using established methods^48,49^.

### RDC data analysis

Residual dipolar couplings, *D*, were measured as the difference between the isotropic and anisotropic splittings in the ^15^N dimension of HSQC and TROSY spectra for the ribosome, and IPAP ^1^H-coupled ^15^N-HSQC spectra for isolated bL12 (Supplementary Table S2). Uncertainties, *σ*_*D*_, were propagated using standard methods from the uncertainty in peak positions, determined from the signal-to-noise ratio (SN) and ^15^N linewidth (LW), *σ*_*v*_ = LW*/*2SN. Initial data analysis was performed by fitting the measured RDCs to the available NMR-derived structure of the bL12 protein (PDB code 1rqu^35^), as implemented by the SVD (singular-value-decomposition) routine in the program PALES^36^, in order to derive the alignment tensor (*A*) and the corresponding set of back-calculated RDCs. Orientations of alignment tensors are relative to the PDB frame of the template structure. To be able to restrict the analysis of RDCs to the structure and orientational probability distribution of the domain, but not the local dynamics within the domain, residues with local flexibility (order parameter *S*^2^ < 0.85) or undergoing conformational exchange are excluded from the structure and alignment tensor analysis. In addition, residues whose resonances are overlapped in the spectra and residues with experimental uncertainties *σ*_*D*_ > 5 Hz are excluded.

### Structure refinement

The template structure for the CTD of bL12 was refined using Xplor-NIH^50^. The refinement was implemented in a simulating- annealing molecular-dynamics simulation by introducing an additional term to the force-field whose energy depends on the difference between the experimental RDC and that back-calculated from the structure. In the refinement, only the orientation of the secondary-structure elements (individual *α*-helices and *β*-strands) is optimized^51^ and the local structure of the secondary structure elements was fixed using non-crystallographic symmetry (NCS) restraints, and only those RDCs corresponding to rigid N-H vectors within the secondary structure elements were included as restraints. The values for the magnitude and rhombicity of the alignment tensor for back-calculation of the RDCs were taken as those of the best-fit alignment tensor derived using the template structure. The orientation of the alignment tensor is allowed to float during the refinement. The RDC restraints were implemented as soft-square-well susceptibility anisotropy (SANI) restraints, with the width of the according to the experimental uncertainty (*σ*_*D*_) of each measured RDC. Euler angles for rotation of the alignment tensor from the PDB frame into the principal axis frame before and after structural refinement of bL12 free and bL12 ribo CTD are listed in Supplementary Table S3. The five components of the irreducible tensor representations of the alignment tensors and their general magnitudes are shown in Supplementary Table S4.

## Supplementary information

Supplementary Informations
