# Supplementary material for: Probing the dynamic stalk region of the ribosome using solution NMR
Source: Sci Rep. 2019 Sep 19;9:13528. doi: 10.1038/s41598-019-49190-1 (PMC6753160; doi:10.1038/s41598-019-49190-1)
Supplement: Supplementary file 1 — Supplementary Informations [file 41598_2019_49190_MOESM1_ESM.docx]

**Supplementary information**

**Probing the dynamic stalk region of the ribosome using solution NMR**

Xiaolin Wang, John P. Kirkpatrick, Hélène M. M. Launay, Alfonso de Simone, Daniel Häussinger, Christopher M. Dobson, Michele Vendruscolo, Lisa D. Cabrita, Christopher A. Waudby and John Christodoulou

This document contains:

Supplementary Figure 1: Deuterium splitting spectra.

Supplementary Figure 2: Comparison between IPAP and HSQC/TROSY approach.

Supplementary Figure 3: ^1^H STE diffusion of isotropic and aligned 70S ribosomes.

Supplementary Figure 4: Correlation plots of RDCs.

Supplementary Figure 5: Chemical shift changes between bL12_free_ wild-type and cysteine variants.

Supplementary Figure 6: ^1^H,^15^N-HSQC spectra for bL12_free_ mutants.

Table S1: Table of quality and alignment tensor parameters after SVD fitting.

Table S2: Table of RDC D_NH_ values.

Table S3: Table of Euler angles for rotation of alignment tensors from the PDB frame to the respective principal axis frames.

Table S4: Table of components of the irreducible tensor representations and general magnitudes of the alignment tensors.


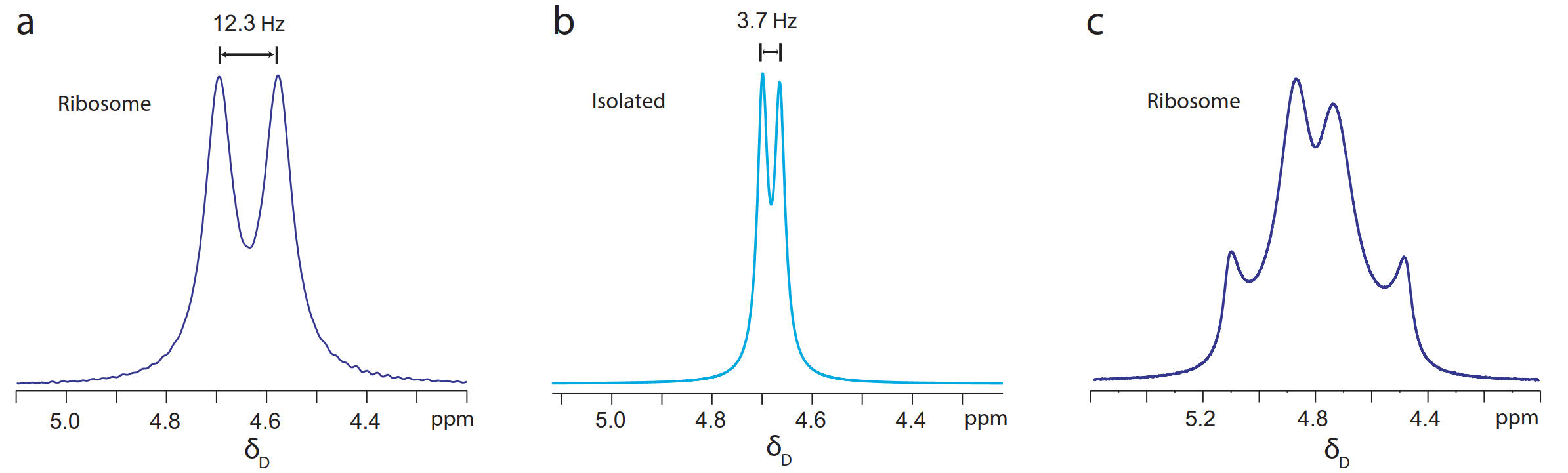


Supplementary Figure 1: 1D ^2^H spectra of 70S ribosome (a) and bL12_free_ (b) aligned in Pf1 phage on the 700MHz spectrometer, showing splitting of 12.3 Hz and 3.7 Hz at 298 K. (c): 1D ^2^H spectra of 70S ribosome aligned in C_12_E_5_ PEG and hexanol medium (Rückert and Otting, 2000).


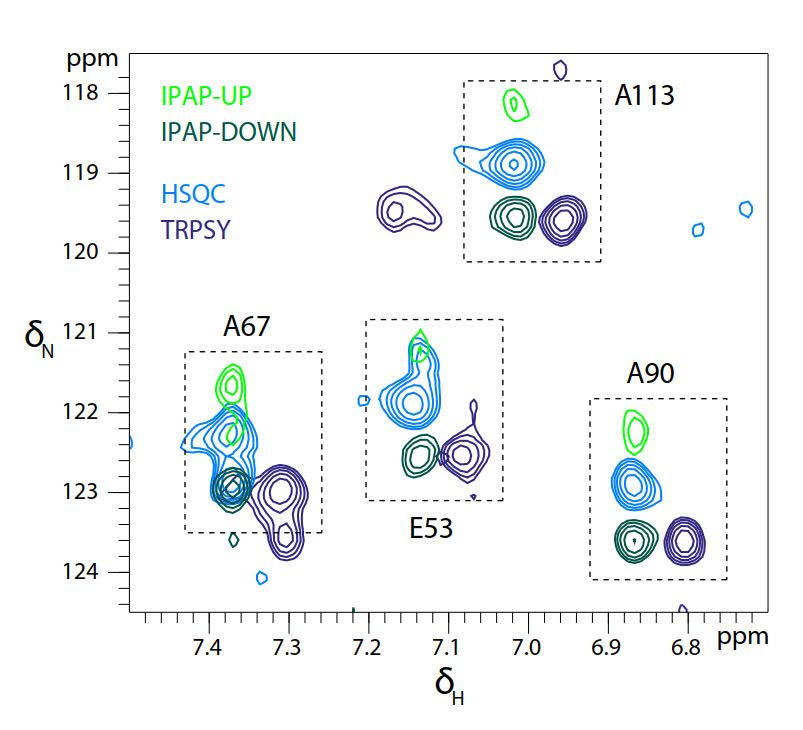


Supplementary Figure 2: Comparison between IPAP-^15^N-HSQC spectra (light and dark green) and ^15^N-HSQC and ^15^N-TROSY spectra (light and dark blue) acquired on 70S ribosomes under the same conditions. The spectra were recorded for the same total experiment time and contoured at the same level (4$\boldsymbol{\times}$RMS noise). A selected region is displayed, containing cross-peaks from residue E53, A67, A90 and A113. The sensitivity of the IPAP-UP spectrum being lower, resulting in higher uncertainty on the RDC measurement in the IPAP strategy than that using the HSQC-TROSY strategy.


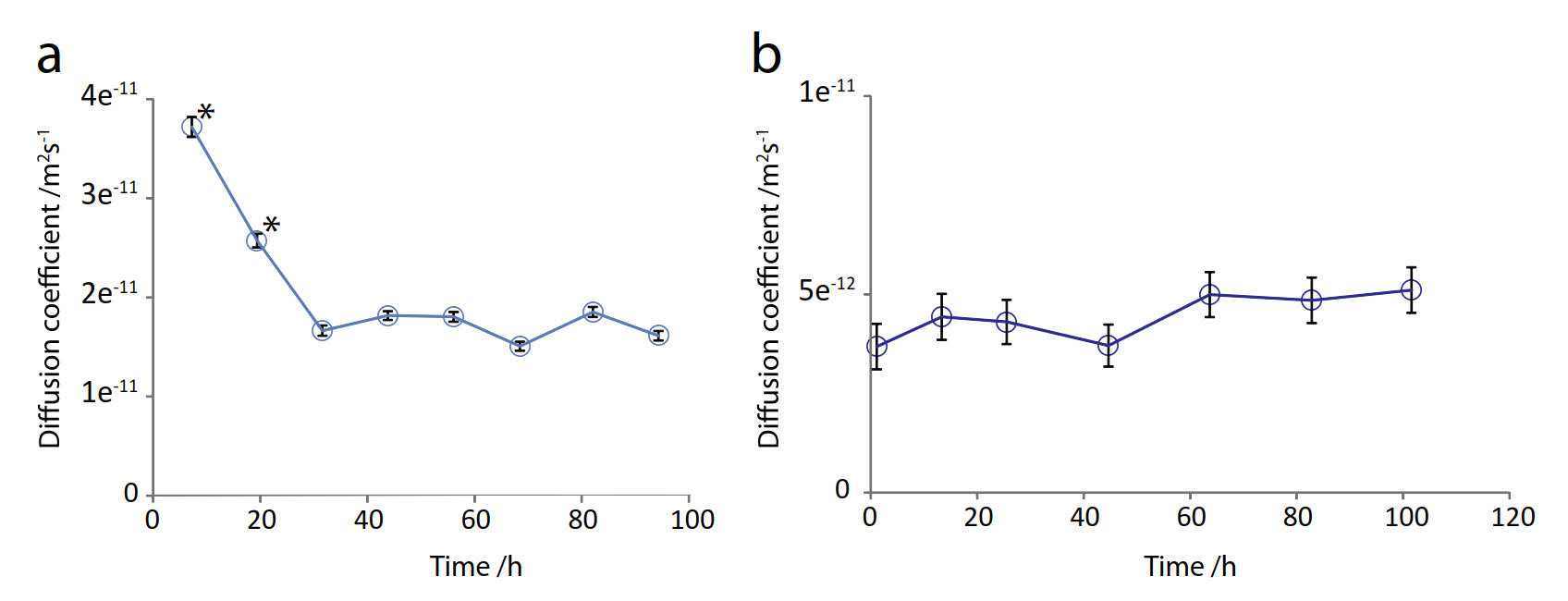


Supplementary Figure 3: Plot of ^1^H STE diffusion measurements of 70S ribosome samples used for RDC data measurements for (a) isotropic sample of ^15^N labeled ribosome and (b) phage aligned sample of ^15^N labeled ribosome. Points labelled with (*) contain systematic errors arising from poor water suppression and therefore a poor baseline, which was corrected in later measurements.


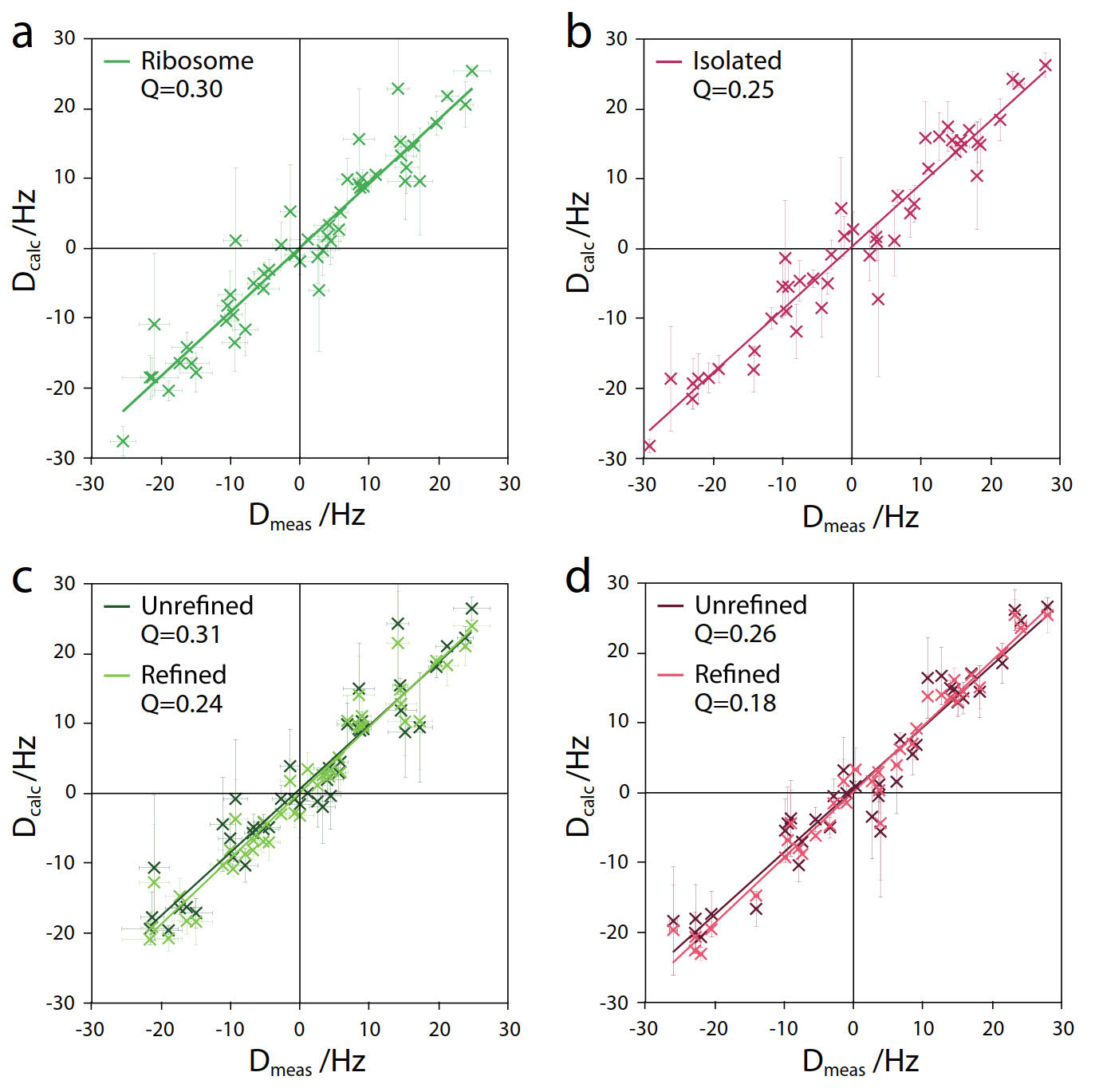


Supplementary Figure 4: Correlation plots between the measured and back-calculated RDCs for the CTD of bL12_ribo_ (a,c) and bL12_free_ (b,d) aligned in Pf1 phage. The x-error bars represent the uncertainties in the measured RDCs, as estimated from the signal-to-noise ratios and linewidths of the spectral peaks. The y-error bars correspond to the uncertainties in the back-calculated RDCs, as derived from the Monte-Carlo “mcDc” routine in the PALES software


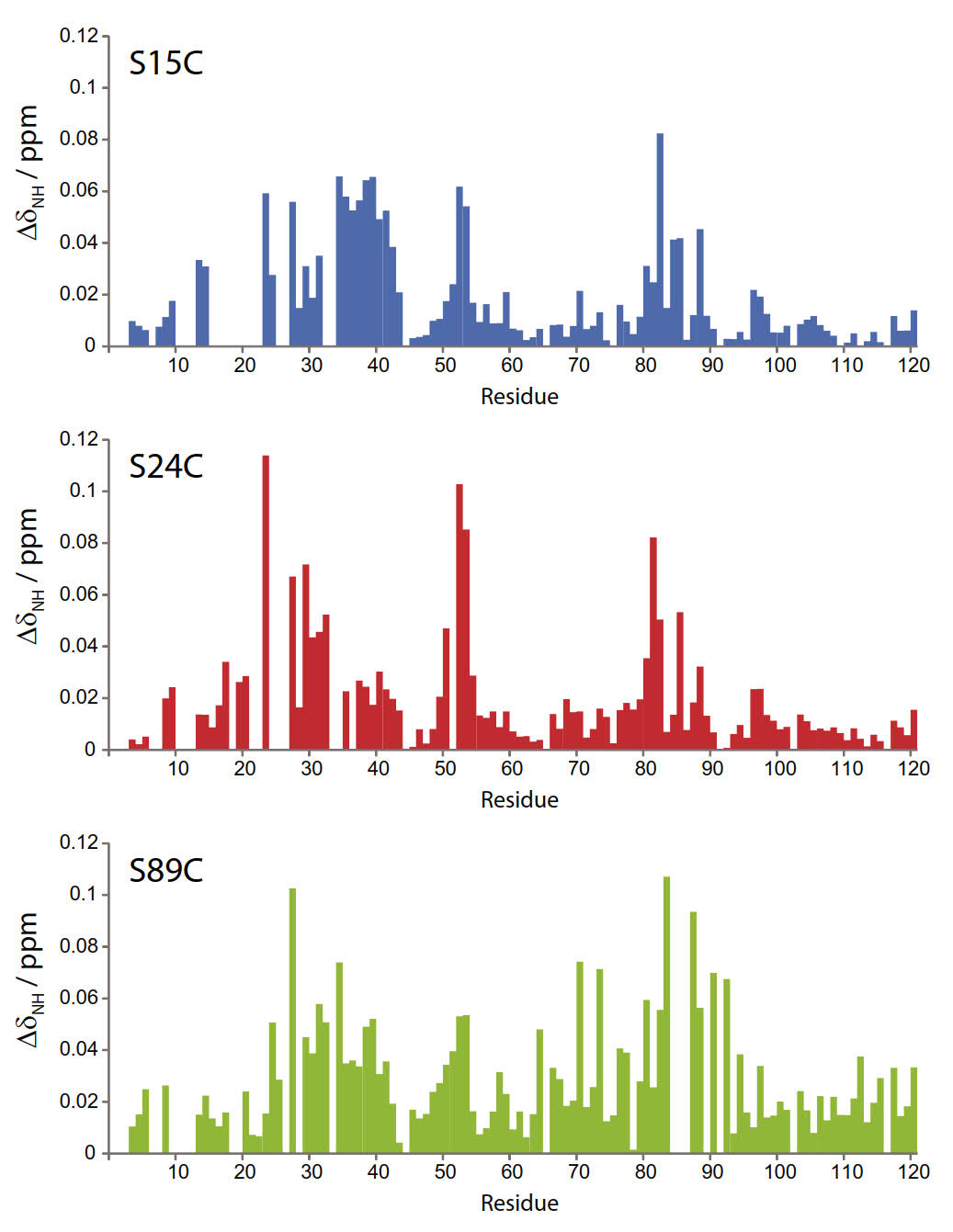


Supplementary Figure 5: ΔδNH chemical shift changes between bL12_free_ wild-type and cysteine variants expressed as ΔδNH=[Δδ_H_^2^+(Δδ_N_/5)^2^]^1/2^.


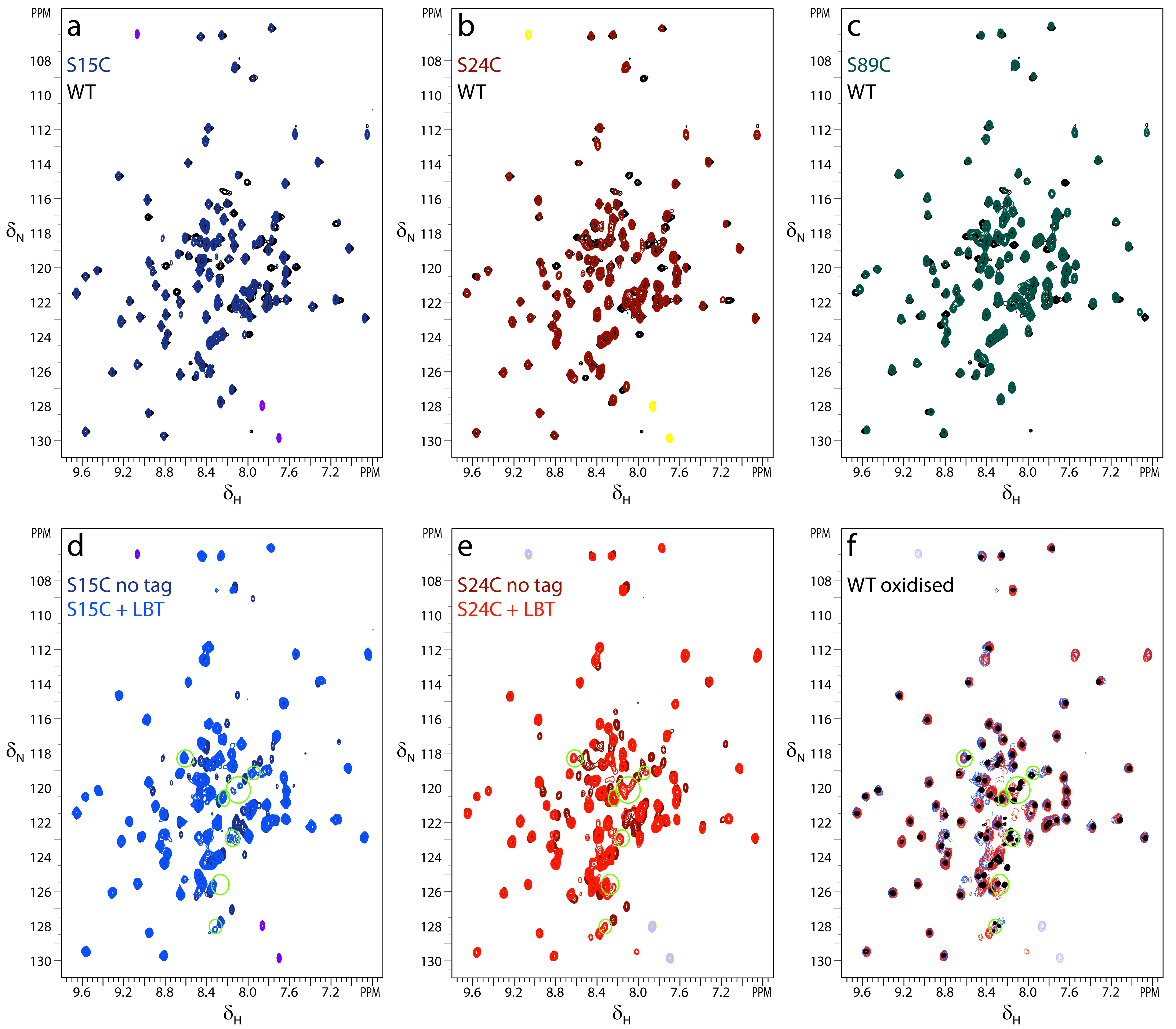


Supplementary Figure 6: (a-c) Overlay of ^1^H,^15^N-HSQC spectra for bL12_free_ mutants S15C, S24C and S89C with wild-type protein. (d) Overlay of S15C before and after reaction with LBT tag. (e) Overlay of S24C before and after reaction with tag. (f) Overlay of S15C-LBT and S24C-LBT with wild-type protein being oxidized with H_2_O_2_. Green circles (panels d-f) highlight signals appeared after reaction with LBT tag.

|  | Parameters | N | RMS_err_  / Hz | RMSD / Hz | Q | D_a_^NH^ / Hz | σD_a_^NH^ / Hz | R | σR |
| --- | --- | --- | --- | --- | --- | --- | --- | --- | --- |
| 1 | bL12_free_  (all residues) | 47 | 0.35 | 3.8 | 0.25 | -20.5 | 2.1 | 0.204 | 0.074 |
| 2 | bL12_ribo_  (all residues) | 53 | 1.83 | 3.9 | 0.30 | -18.1 | 1.1 | 0.303 | 0.047 |
| 3 | bL12_free_ (secondary structure, before refinement) | 37 | 0.30 | 3.7 | 0.26 | -20.2 | 2.0 | 0.262 | 0.081 |
| 4 | bL12_free_ (secondary structure, after refinement) | 37 | 0.30 | 2.5 | 0.18 | -20.1 | 1.7 | 0.219 | 0.065 |
| 5 | bL12_ribo_ (secondary structure, before refinement) | 44 | 1.86 | 3.9 | 0.31 | -18.1 | 1.3 | 0.309 | 0.049 |
| 6 | bL12_ribo_ (secondary structure, after refinement) | 44 | 1.86 | 3.0 | 0.24 | -17.4 | 1.0 | 0.266 | 0.039 |
| 7 | bL12_free_ CTD^2-linker^ | 40 | 0.25 | 0.2 | 0.92 | -0.01 | 0.19 | 0.338 | 7.38 |
| 8 | bL12_free_ NTD^1-linker^ | 13 | 2.11 | 1.5 | 0.58 | 1.16 | 2.06 | 0.289 | 0.885 |
| 9 | bL12_free_ CTD^self^ | 25 | 0.18 | 1.8 | 0.15 | -16.6 | 0.92 | 0.192 | 0.062 |

Table S1: Table of quality and alignment tensor parameters after SVD fitting of measured N-H RDCs to bL12 structure for bL12_ribo_ and bL12_free_ aligned in bacteriophage (rows 1-6) and isolated bL12 aligned by paramagnetic LBTs (rows 7-9). N is the number of RDCs measured, RMS_err_ is the root-mean-square measurement error (uncertainty), RMSD is the root-mean-square-deviation between the measured and back-calculated RDCs. Q is the quality factor, which is a magnitude-independent quality factor that describes the agreement between the measured and the back-calculated RDCs, D_a_^NH^ is the axial component of the alignment a tensor scaled for the N-H coupling and R is the rhombicity of the alignment tensor. σD_a_^NH^ and σR are the uncertainties in D_a_^NH^ and R, respectively.

|  |  | bL12_free_ phage | | bL12_ribo_ phage | | bL12_free_ LBT mix label | | bL12_free_ LBT CTD^self^ | |
| --- | --- | --- | --- | --- | --- | --- | --- | --- | --- |
| Residue | | D_NH_ | **σ** | D_NH_ | **σ** | D_NH_ | **σ** | D_NH_ | **σ** |
| 1 | SER |  |  |  |  |  |  |  |  |
| 2 | ILE |  |  |  |  |  |  |  |  |
| 3 | THR | 0.89 | 0.37 |  |  |  |  |  |  |
| 4 | LYS | 0.34 | 0.35 |  |  | 2.28 | 1.41 |  |  |
| 5 | ASP | 0.52 | 0.18 |  |  |  |  |  |  |
| 6 | GLN |  |  |  |  |  |  |  |  |
| 7 | ILE | 1.30 | 0.31 |  |  |  |  |  |  |
| 8 | ILE | -0.28 | 0.21 |  |  | 0.70 | 1.52 |  |  |
| 9 | GLU | 0.56 | 0.17 |  |  | 1.54 | 1.41 |  |  |
| 10 | ALA |  |  |  |  |  |  |  |  |
| 11 | VAL |  |  |  |  | 5.63 | 2.22 |  |  |
| 12 | ALA |  |  |  |  |  |  |  |  |
| 13 | ALA | 1.56 | 0.18 |  |  | -1.26 | 1.19 |  |  |
| 14 | MET | 0.21 | 0.16 |  |  | 0.56 | 1.12 |  |  |
| 15 | SER | -0.42 | 0.22 |  |  |  |  |  |  |
| 16 | VAL | 2.14 | 0.36 |  |  |  |  |  |  |
| 17 | MET | 1.26 | 0.16 |  |  | 0.87 | 1.60 |  |  |
| 18 | ASP |  |  |  |  |  |  |  |  |
| 19 | VAL |  |  |  |  |  |  |  |  |
| 20 | VAL | 2.23 | 0.25 |  |  | 3.12 | 3.05 |  |  |
| 21 | GLU | 1.82 | 0.18 |  |  | 1.13 | 2.24 |  |  |
| 22 | LEU | 2.51 | 0.17 |  |  |  |  |  |  |
| 23 | ILE | 1.95 | 0.25 |  |  | -0.98 | 1.63 |  |  |
| 24 | SER | 1.96 | 0.21 |  |  |  |  |  |  |
| 25 | ALA | 1.81 | 0.17 |  |  | -2.16 | 1.72 |  |  |
| 26 | MET |  |  |  |  | -3.38 | 3.83 |  |  |
| 27 | GLU | 0.94 | 0.27 |  |  |  |  |  |  |
| 28 | GLU |  |  |  |  |  |  |  |  |
| 29 | LYS | 2.42 | 0.41 |  |  | -4.49 | 2.58 |  |  |
| 30 | PHE | 2.10 | 0.45 |  |  |  |  |  |  |
| 31 | GLY | 0.58 | 0.31 |  |  |  |  |  |  |
| 32 | VAL |  |  |  |  |  |  |  |  |
| 33 | SER |  |  |  |  |  |  |  |  |
| 34 | ALA | 1.45 | 0.40 |  |  |  |  |  |  |
| 35 | ALA | 0.68 | 0.30 |  |  |  |  |  |  |
| 36 | ALA | -0.17 | 0.22 |  |  |  |  |  |  |
| 37 | ALA | -0.54 | 0.18 |  |  |  |  |  |  |
| 38 | VAL | -1.29 | 0.11 |  |  |  |  |  |  |
| 39 | ALA | -0.40 | 0.18 |  |  |  |  |  |  |
| 40 | VAL | -0.86 | 0.09 | 1.13 | 2.66 |  |  |  |  |
| 41 | ALA | -1.27 | 0.17 |  |  |  |  |  |  |
| 42 | ALA | -0.99 | 0.17 |  |  |  |  |  |  |
| 43 | GLY | -1.02 | 0.12 | -6.52 | 1.41 |  |  |  |  |
| 44 | PRO |  |  |  |  |  |  |  |  |
| 45 | VAL | -0.67 | 0.05 | -3.83 | 0.59 |  |  |  |  |
| 46 | GLU | 0.33 | 0.09 | -4.82 | 0.79 |  |  |  |  |
| 47 | ALA | 0.19 | 0.10 | -2.59 | 0.71 |  |  |  |  |
| 48 | ALA | 2.22 | 0.09 | -0.40 | 0.46 |  |  |  |  |
| 49 | GLU | 4.27 | 0.07 | 2.73 | 0.49 |  |  |  |  |
| 50 | GLU | 6.19 | 0.11 | 4.60 | 0.53 |  |  |  |  |
| 51 | LYS | 13.42 | 0.16 | 5.64 | 0.93 |  |  |  |  |
| 52 | THR | 16.11 | 1.76 |  |  |  |  |  |  |
| 53 | GLU | -17.11 | 0.42 | -14.08 | 2.21 |  |  |  |  |
| 54 | PHE | -7.94 | 0.23 | -7.86 | 1.82 |  |  | 23.09 | 0.10 |
| 55 | ASP | -14.09 | 0.33 | -17.29 | 2.07 | 0.05 | 0.27 | 9.09 | 0.10 |
| 56 | VAL | 8.47 | 0.32 | 3.94 | 2.36 | 0.04 | 0.24 |  |  |
| 57 | ILE | -3.44 | 0.24 | -5.17 | 1.48 | 0.24 | 0.27 |  |  |
| 58 | LEU | 24.11 | 0.83 | 20.34 | 5.32 | -0.13 | 0.36 |  |  |
| 59 | LYS | 27.95 | 0.42 | 24.81 | 2.66 | -0.50 | 0.30 |  |  |
| 60 | ALA | 13.91 | 0.15 | 21.25 | 1.62 | -0.06 | 0.17 |  |  |
| 61 | ALA |  |  | 17.38 | 5.00 |  |  |  |  |
| 62 | GLY | 11.09 | 0.14 | 10.93 | 0.83 |  |  | 14.91 | 0.11 |
| 63 | ALA | 3.28 | 0.17 | 6.20 | 1.15 |  |  |  |  |
| 64 | ASN | -36.01 | 0.81 | -30.66 | 3.60 |  |  |  |  |
| 65 | LYS |  |  |  |  |  |  |  |  |
| 66 | VAL | 10.66 | 0.18 | 8.70 | 1.26 | -0.25 | 0.24 | -1.09 | 0.07 |
| 67 | ALA | 16.96 | 0.22 | 8.98 | 1.34 | 0.14 | 0.17 |  |  |
| 68 | VAL |  |  | 15.19 | 2.48 | -0.04 | 0.26 | 13.14 | 0.07 |
| 69 | ILE | 0.24 | 0.43 | 1.14 | 2.53 | -0.20 | 0.40 | -0.40 | 0.10 |
| 70 | LYS | 12.65 | 0.20 | 9.17 | 1.13 | 0.39 | 0.32 | 0.05 | 0.07 |
| 71 | ALA | 18.17 | 0.23 | 17.30 | 1.84 |  |  | 15.94 | 0.07 |
| 72 | VAL | 3.54 | 0.26 | 4.46 | 1.50 |  |  |  |  |
| 73 | ARG | 2.62 | 0.26 | 3.32 | 1.82 | -0.11 | 0.37 | -3.55 | 0.14 |
| 74 | GLY | 14.51 | 0.26 | 6.86 | 1.48 |  |  |  |  |
| 75 | ALA |  |  | 8.53 | 2.25 | -0.29 | 0.18 |  |  |
| 76 | THR | -13.97 | 0.33 | -9.39 | 1.87 |  |  |  |  |
| 77 | GLY | 18.56 | 0.28 | 15.36 | 1.67 |  |  |  |  |
| 78 | LEU | -9.41 | 0.57 | -10.42 | 1.48 |  |  |  |  |
| 79 | GLY | 15.75 | 1.25 | -2.15 | 7.95 |  |  |  |  |
| 80 | LEU | -20.57 | 0.19 | -14.95 | 2.36 |  |  |  |  |
| 81 | LYS |  |  | -6.66 | 0.91 |  |  |  |  |
| 82 | GLU | 3.67 | 0.29 | 0.04 | 2.02 | 0.14 | 0.23 |  |  |
| 83 | ALA | -22.84 | 0.37 | -21.28 | 1.68 | 0.08 | 0.38 |  |  |
| 84 | LYS |  |  |  |  | 0.40 | 0.39 |  |  |
| 85 | ASP | -2.90 | 0.16 | 0.78 | 4.43 | -0.12 | 0.28 |  |  |
| 86 | LEU |  |  | -5.23 | 2.24 |  |  |  |  |
| 87 | VAL | -22.92 | 0.28 | -18.90 | 1.93 |  |  |  |  |
| 88 | GLU |  |  | -10.02 | 1.68 | -0.12 | 0.34 |  |  |
| 89 | SER | 15.80 | 0.16 | 14.49 | 1.06 | 0.01 | 0.30 |  |  |
| 90 | ALA | -29.12 | 0.36 | -25.48 | 1.82 |  |  |  |  |
| 91 | PRO |  |  |  |  |  |  |  |  |
| 92 | ALA |  |  | 14.15 | 1.61 | 0.09 | 0.26 |  |  |
| 93 | ALA | 23.23 | 0.19 | 23.86 | 1.14 | -0.18 | 0.17 | -12.36 | 0.68 |
| 94 | LEU | -9.91 | 0.29 | -11.09 | 1.87 | 0.04 | 0.26 | -11.16 | 0.27 |
| 95 | LYS | -5.52 | 0.16 | -6.75 | 1.18 | 0.02 | 0.15 | 10.85 | 0.14 |
| 96 | GLU | -4.65 | 0.50 | -5.03 | 1.80 |  |  |  |  |
| 97 | GLY | 6.67 | 0.23 | 4.23 | 2.17 | -0.04 | 0.22 | 19.54 | 0.09 |
| 98 | VAL | 15.00 | 0.21 | 8.49 | 1.33 | 0.25 | 0.17 | 22.17 | 0.08 |
| 99 | SER | -19.14 | 0.39 | -15.60 | 2.52 |  |  | 12.74 | 0.09 |
| 100 | LYS | -22.07 | 0.28 | -16.28 | 2.18 | -0.06 | 0.21 | -10.19 | 0.12 |
| 101 | ASP | -1.05 | 0.21 | -2.68 | 1.24 | -0.13 | 0.15 | -3.46 | 0.08 |
| 102 | ASP |  |  |  |  |  |  |  |  |
| 103 | ALA |  |  | -24.79 | 3.94 | -0.16 | 0.19 | -13.46 | 0.13 |
| 104 | GLU | -9.55 | 0.28 | -9.25 | 1.74 | 0.02 | 0.16 | -3.35 | 0.07 |
| 105 | ALA |  |  |  |  |  |  |  |  |
| 106 | LEU | 3.85 | 0.17 | -20.98 | 2.16 | -0.60 | 0.18 | -12.93 | 0.19 |
| 107 | LYS |  |  |  |  | 0.02 | 0.18 | -9.04 | 0.15 |
| 108 | LYS | -1.48 | 0.22 | -1.38 | 1.47 | 0.29 | 0.15 |  |  |
| 109 | ALA |  |  | -0.74 | 1.40 | -0.15 | 0.13 |  |  |
| 110 | LEU | -26.05 | 0.57 | -21.58 | 4.05 | -0.33 | 0.23 |  |  |
| 111 | GLU | -7.45 | 0.21 | -4.43 | 1.68 | -0.05 | 0.20 |  |  |
| 112 | GLU |  |  | 5.87 | 1.10 |  |  |  |  |
| 113 | ALA | -9.09 | 0.21 | -9.60 | 1.27 | 0.03 | 0.17 | -12.07 | 0.10 |
| 114 | GLY | -4.30 | 0.19 | 2.76 | 1.40 |  |  | -7.07 | 0.09 |
| 115 | ALA | 18.08 | 0.16 | 16.39 | 0.98 |  |  |  |  |
| 116 | GLU |  |  | 14.60 | 2.23 | -0.29 | 0.23 | -13.09 | 0.10 |
| 117 | VAL | 21.42 | 0.18 | 19.69 | 1.17 | -0.05 | 0.21 | -15.77 | 0.09 |
| 118 | GLU | 6.18 | 0.39 | 2.56 | 2.19 | 0.04 | 0.22 |  |  |
| 119 | VAL | 9.04 | 0.11 | 5.62 | 0.96 | 0.09 | 0.16 |  |  |
| 120 | LYS | -11.51 | 0.36 | -10.61 | 1.74 |  |  |  |  |

Table S2: RDC D_NH_ values of bL12_free_ and bL12_ribo_ CTD aligned by phage and bL12_free_ LBT.

| **Euler Angles** |  | **ALPHA** | **BETA** | **GAMMA** |
| --- | --- | --- | --- | --- |
| **bL12_free_ (Before)** | 1 | 215.71 | 118.62 | 225.56 |
|  | 2 | 35.71 | 118.62 | 225.56 |
|  | 3 | 324.29 | 61.38 | 45.56 |
|  | 4 | 144.29 | 61.38 | 45.56 |
| **bL12_free_ (After)** | 1 | 218.25 | 118.02 | 226.9 |
|  | 2 | 38.25 | 118.02 | 226.9 |
|  | 3 | 321.75 | 61.98 | 46.9 |
|  | 4 | 141.75 | 61.98 | 46.9 |
| **bL12_ribo_ (Before)** | 1 | 58.88 | 114.95 | 228.2 |
|  | 2 | 238.88 | 114.95 | 228.2 |
|  | 3 | 121.12 | 65.05 | 48.2 |
|  | 4 | 301.12 | 65.05 | 48.2 |
| **bL12_ribo_ (After)** | 1 | 60.08 | 114.87 | 227.93 |
|  | 2 | 240.08 | 114.87 | 227.93 |
|  | 3 | 119.92 | 65.13 | 47.93 |
|  | 4 | 299.92 | 65.13 | 47.93 |

**Table S3:** Euler angles for rotation of the alignment tensor from the PDB frame into the principal axis frame before and after structural refinement of bL12_free_ and bL12_ribo_ CTD aligned by phage. The angles correspond to the Euler convention of clockwise rotations about the three independent axes *z* (angle ALPHA), *y*’ (angle BETA) and *z’*’ (angle GAMMA). Four different Euler angles are reported in each case due to the fourfold degeneracy of alignment tensors.

|  | A0 | A1R | A1I | A2R | A2I | Gen_Mag |
| --- | --- | --- | --- | --- | --- | --- |
| bL12_free_ (Before) | 3.31E-04 | -7.68E-04 | -1.30E-03 | -1.70E-04 | -1.49E-03 | 3.03E-03 |
| bL12_free_ (After) | 4.22E-04 | -7.80E-04 | -1.29E-03 | -6.66E-05 | -1.48E-03 | 3.01E-03 |
| bL12_ribo_ (Before) | 8.70E-04 | -6.03E-04 | -1.29E-03 | -5.18E-05 | -1.24E-03 | 2.81E-03 |
| bL12_ribo_ (After) | 8.11E-04 | -6.16E-04 | -1.17E-03 | -2.99E-05 | -1.18E-03 | 2.63E-03 |

Table S4: The five components of the irreducible tensor representations (Sass et al., 1999) of the alignment tensors and their general magnitudes before and after structural refinement of bL12_free_ and bL12_ribo_ CTD aligned by phage.
